# Longitudinal Study Depicting Differences in Complementary Feeding and Anthropometric Parameters in Late Preterm Infants up to 2 Years of Age

**DOI:** 10.3390/nu13030982

**Published:** 2021-03-18

**Authors:** María Gómez-Martín, David Herrero-Morín, Gonzalo Solís, Marta Suarez, Nuria Fernández, Silvia Arboleya, Miguel Gueimonde, Sonia González

**Affiliations:** 1 Department of Functional Biology, University of Oviedo, 33006 Oviedo, Spain; gomezmarmaria@uniovi.es; 2 Diet, Microbiota and Health Group, Instituto de Investigación Sanitaria del Principado de Asturias (ISPA), 33011 Oviedo, Spain; nuriajmhd@gmail.com (N.F.); silvia.arboleya@ipla.csic.es (S.A.); mgueimonde@ipla.csic.es (M.G.); 3 Pediatrics Service, Centro Atención Primaria Infiesto, SESPA, 33530 Infiesto, Spain; herrerojose@uniovi.es; 4 Pediatrics Service, Hospital Universitario Central de Asturias, SESPA, 33011 Oviedo, Spain; gonzalosolissanchez7@gmail.com (G.S.); msr1070@hotmail.com (M.S.); 5 Pediatrics Research Group, Instituto de Investigación Sanitaria del Principado de Asturias (ISPA), 33011 Oviedo, Spain; 6 Pediatrics Service, Hospital de Cabueñes, SESPA, 33394 Gijón, Spain; 7 Department of Microbiology and Biochemistry of Dairy Products, Instituto de Productos Lácteos de Asturias (IPLA-CSIC), 33300 Villaviciosa, Spain

**Keywords:** preterm, complementary feeding, vitamin D, protein intake

## Abstract

Ensuring the nutritional demands of preterm (PT) infants during complementary feeding could contribute significantly to the infants’ long-term health and development. However, the dietary guidelines for complementary feeding in PT are scarce. Thus, describing dietary intake and identifying nutritional targets for these infants could be of great interest. The aim of this study is to assess the food intake and anthropometric parameters in a Mediterranean infant cohort from 6 to 24 months and to identify nutritional targets especially focused on late preterm infants. This is a longitudinal prospective study analyzing information from administered questionnaires about general characteristics and food frequency consumption in 115 infants (20 PT (32 to 36 gestational weeks), 95 full-term (FT)) at 6, 12 and 24 months of age. Results show that the differences in the prevalence of underweight observed in PT infants vs. FT infants are maintained for up to 6 months of age but disappear at 12 and 24 months. The age of inclusion of new foods and the average intake of the main food groups was not different from that of FTs. Although protein intake at 6 months was directly correlated with weight gain and growth in FT, these associations were not observed in PT. At the nutritional level, the low intake of vitamin D in preterm infants is noteworthy. These findings may be useful when designing new intervention strategies for this population group.

## 1. Introduction

Prematurity is a major cause of neonatal morbidity and mortality worldwide [1,2,3,4]. It is estimated that approximately one in ten births in Spain takes place before the 37th week of gestation, the highest such rate within the European Union. If proper nutrition is required for the maintenance of health at all life stages, this factor is particularly important in preterm infants (PT) to ensure not only fulfilling nutritional requirements, but also an optimum growth and the correct development of the immune and nervous system [5,6].

The World Health Organization (WHO), along with the European Society for Pediatric Gastroenterology Hepatology and Nutrition [7] (ESPGHAN), recommends exclusive breastfeeding until 6 months of age for full-term (FT) healthy babies [8]. Subsequently, complementary feeding (CF), defined as “the introduction of nutritive liquids, different of breast milk, or solids, when breast milk is no longer sufficient to meet all nutritional requirements of infants”, represents a gradual transition to the family diet [7]. At this point, several authors have highlighted the need to define both the optimal age for solid introduction in PT and the food portions necessary to ensure that nutritional, metabolic and growth requirements are met [9,10]. Theoretically, the maturation of the digestive tract and the renal system, necessary for the absorption and metabolism of the nutrients contained in the complementary diet, takes place at around 4 months in FT infants [11]. However, previous studies have evidenced that a high percentage of PT infants consumed complementary food before this age [12,13]. The weaning process before 17 weeks in PT infants has been associated with an increased risk of allergy or obesity in the long term [13,14]. On the contrary, the delay of solid food introduction until 6 months could lead to deficiencies in terms of energy and protein intake as well as in essential components such as iron, copper, zinc, calcium, phosphorus and vitamin D [15], whose stores at birth may have been compromised in these infants [16]. Since essential nutrients are involved in a large number of functions during early life, the consequences of their deficiency in PT neonates may be severe. For example, anemia is common in preterm infants in the first 4 months of age, which may cause problems in erythropoiesis and neurological development [17]. There is controversy about the impact of high protein consumption on growing parameters. However, some authors have found that greater protein intakes in preterm babies with basal weights between 900–1500 g would increase weight, length and head circumference [18], while the addition of lipids together with the protein supply only promoted weight gain and an increase in adiposity [18]. Moreover, newborns, especially preterm infants, are particularly vulnerable to oxidative damage because of their weak antioxidant systems [19]. Therefore, the intake of some bioactive compounds, such as (poly)phenols, with a well-proven antioxidant effect [20], could be particularly useful in the prevention of oxidative-stress-related pathologies.

Further studies are necessary to decipher the impact of weaning practices on short- and long-term health outcomes in order to provide the basis for the development of appropriate nutritional recommendations. Based on the available evidence, it is proposed that there may be differences in complementary feeding and nutritional intake between term and preterm infants. Thus, our aim is to identify both qualitative and quantitative nutritional targets in pre-term babies that may be useful to construct specific nutritional recommendations during the complementary feeding period.

## 2. Subjects and Methods

### 2.1. Design and Sample Recruitment

**Figure 1:**
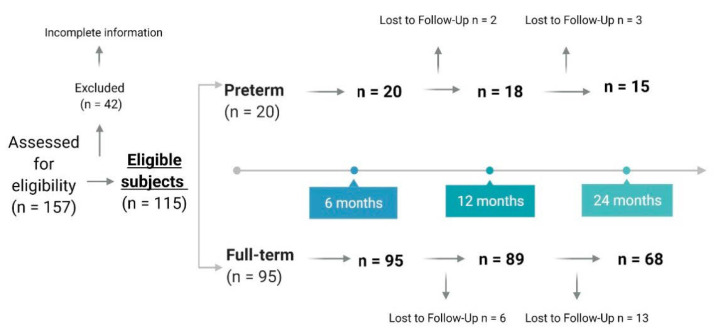
Scheme of participant enrollment and study progress.

The analyses present in this work have been carried out in a sub-cohort from the longitudinal prospective project Early-MicroHealth “Impact of early life diet on microbiome development and later health”. The sample population consisted of 115 babies, 20 late PT (32–36 gestational weeks) and 95 FT (37–40 post conceptual weeks) (Figure 1). Preterm newborns were recruited, in the days subsequent to birth, by the neonatal specialists at Central University Hospital of Asturias (HUCA). FT newborns were enrolled through the primary care pediatric service in the first visit to the consultation, which takes place in the first 10–15 days of the infant’s life. Parents were informed in writing about the contents, procedures and aims of the study, as well as about the option to withdraw from the study at any time. In all cases, written informed consent was obtained before enrolment. Of the initial recruited sample, 42 children failed to start the study by not completing all the steps of the study. Subsequently, for this analysis, data from all children who had not completed the dietary questionnaire at 6 months of age were discarded.

The project has been evaluated and approved by the Regional Ethics Committee of Clinical Research of Asturias (Ref. 12/16, 3 February 2016) and to the Committee on Bioethics of CSIC (Ref. PCIN-2015-233). The procedures have been performed in accordance with the fundamental principles set out in the Declaration of Helsinki, the Oviedo Bioethics Convention, the Council of Europe Convention on Human Rights and Biomedicine and in the Spanish legislation on bioethics. The Directive 95/46/EC of the European Parliament and the Council of 24 October 1995 on the protection of individuals regarding the processing of personal data and on the free movement of such data were strictly followed.

### 2.2. General Characteristics

At baseline, information on characteristics from mother (gestational age, pre-gestational and at delivery Body Mass Index (BMI), clinical history (chronic diseases, medication use), smoking habit, number of previous pregnancies), delivery (type, use of antibiotics intrapartum, week of gestation) and from neonate (weight and height at birth, gender) were collected.

The nutritional assessment of the sample and some other parameters related to gestation and delivery, relevant to the study, was collected at the time of birth and at 3, 6, 12 and 24 months of age.

### 2.3. Dietary Assessment and Nutritional Data Analysis

Infant’s dietary information was collected by means of a weekly food propensity questionnaire adapted from the Pilot study for Assessment of Nutrient intake and food Consumption Among Kids in Europe (PANCAKE) [21] for the Spanish population, thus including typical foods and traditional regional recipes. In addition, food diaries were designed through an online tool that included detailed dietary information grouped into 11 groups according to European Prospective Investigation into Cancer (EPIC) classification [22] and two extra groups for processed infant food and human breast milk. Groups included oils (vegetable oils and solid fats); vegetables (bulbs, mushrooms, roots, inflorescences, and stem and leaf vegetables); legumes (lentils, chickpeas, beans and peas); fruits (fresh, dried, and canned fruits); potatoes and tubers (potato and sweet potato); cereals and cereal products (bread, pasta, breakfast cereals, flours and grains); meat and meat products (poultry, red meat, processed meat, and others); fish (fish and fish products, crustaceans and mollusks); eggs (eggs and egg products); human breast milk; processed infant products (infant formulas: starter formulas, special starter formulas, follow-up formulas, special follow-up formulas, growing-up milk; infants cereals and infant purees: fruits, fruit and cereals, vegetables, legumes and pasta, meat, fish); milk and dairy products (milk, yogurt, dairy dessert, fresh, mature, and processed cheeses); and sweets and desserts (sweets, cake, biscuits, chocolate, honey, and others).

The questionnaires were completed by the mother, father or caregiver of the child, who received them through their mobile phones. Detailed instructions for completing the food diary were included at the beginning of each category, and the validated picture book developed by PANCAKE consortium was used for portion size estimation according to EU-Menu recommendations [23]. Since the dietary information was collected longitudinally at different times from the time of birth, an adapted version of it was used at 0 and 3 months, due to the absence of complementary feeding. Breastfeeding was collected at all time points both categorically and quantitatively. For the categorical variable, the type of breastfeeding was classified as exclusive, mixed or infant formula. For the quantitative variables, the amount of breast milk consumed was estimated by using the mean values reported in the literature for each stage of age (780 mL for infants under 6 months and 600 mL for infants aged 6–12 months, in the cases of exclusive breastfeeding). For the infant formulas, the volume reported by the parents was used, assuming that the manufacturer’s prescriptions regarding the weight of powdered milk to be dissolved per volume were respected. In the children who received mixed feeding, using the procedure described by Denney et al. and Devaney et al. [24,25], the amount of starter formula consumed per day was measured and the remaining volume of formula consumed per day was assumed to be breast milk.

The consumption of foods was converted into energy and macronutrients using the food composition tables developed by the Centro de Enseñanza Superior de Nutrición Humana y Dietética (CESNID) [26]. Nutritional composition data of human breast milk, infant formula, cereal products and complementary foods (mixed puree or snacks and desserts) was completed from Gómez-Martín et al.’s baby foods composition table [27]. Additionally, detailed information regarding type of protein or carbohydrate consumed was obtained from the food composition tables published by the United States Department of Agriculture (USDA) [28], the content of the major classes and subclasses of (poly)phenol in foods from the Phenol-Explorer Database [29] and the major classes of fiber (soluble and insoluble types) from Marlett et al. [30].

### 2.4. Anthropometric Measures

Child height and weight were recorded to the nearest 0.1 cm and 0.1 kg, respectively, by a pediatric nurse with calibrated and approved material in each of the stages included in the study. Body mass index was calculated as weight, in kilograms, divided by the square of height, in meters, and adjusted by child age and gender. Weight, height and BMI z-scores were calculated relative to WHO Child Growth Standards by using WHO ANTHRO, Software for Calculating Anthropometry, Version 2.0 [31,32,33]. The weight and height gained were calculated by subtracting the weight and height at birth from the weight and height at the time of analysis.

### 2.5. Statistical Analyses

The comparative analysis of diet-related variables across the two groups under study (PT and FT) has been conducted through the IBM SPSS 24.0 (IBM SPSS, Inc., Chicago, IL, USA). The goodness of fit to the normal distribution was checked by means of the Kolmogorov–Smirnov test. Overall, categorical variables were summarized as percentages and continuous ones using means and standard deviations. The Mann–Whitney test, Student *t*-test and two-way ANOVA analysis were used to evaluate differences in continuous variables between PT and FT and chi-squared and Fisher’s analysis for the categorical ones. In addition, Spearman’s rank was used for correlations. GraphPad Prism 8 was used for graphical representations.

Adherence to dietary reference values (DRVs) was calculated using the EFSA (European Food Safety Authority) recommendations for children aged 6 months and 1–3 years [34].

## 3. Results

### 3.1. General Description of the Sample

The basal characteristics between PT and FT neonates are described in Table 1. As expected, significant differences were found in the babies’ weight for age z-score and height for age z-score between groups, and these anthropometric parameters were lower in the PT group. From the maternal variables explored, the prevalence of arterial hypertension at pregnancy, urinary infections and corticoids intrapartum were higher in preterm mothers. In addition, a higher percentage of first-time mothers was found in the PT group.

**Figure 2:**
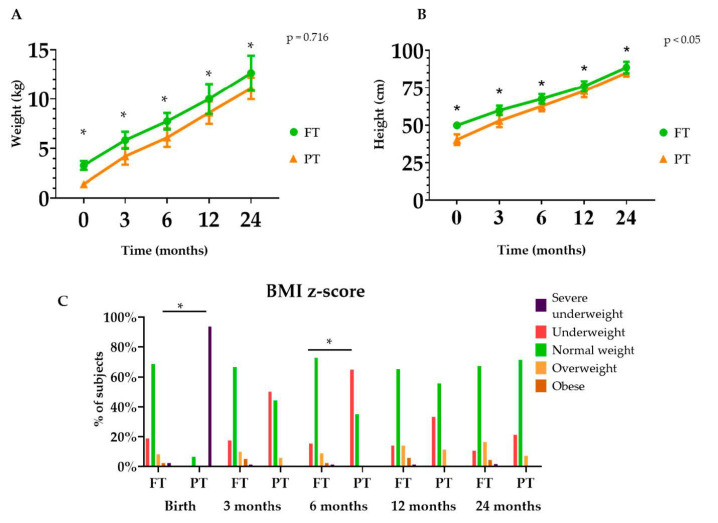
Weight (**A**), height (**B**) and (**C**) BMI z-score during the first 2 years of life between full-term (FT) and preterm (PT) infants. Chi-square was used for intra-group comparisons. * *p* < 0.05. The *p*-values presented in the graph correspond to a two-way ANOVA controlled by time.

In order to expand this information, the growth trend from birth to two years of age in the studied groups is graphically represented in Figure 2. Results show higher weight and height in the group of FT infants across age (Figure 2A,B). The percentage of PT classified as severely underweight and underweight, according to BMI z-score, was higher at birth and at 6 months respectively, in comparison with FT infants (Figure 2C).

### 3.2. Complementary Feeding

**Figure 3:**
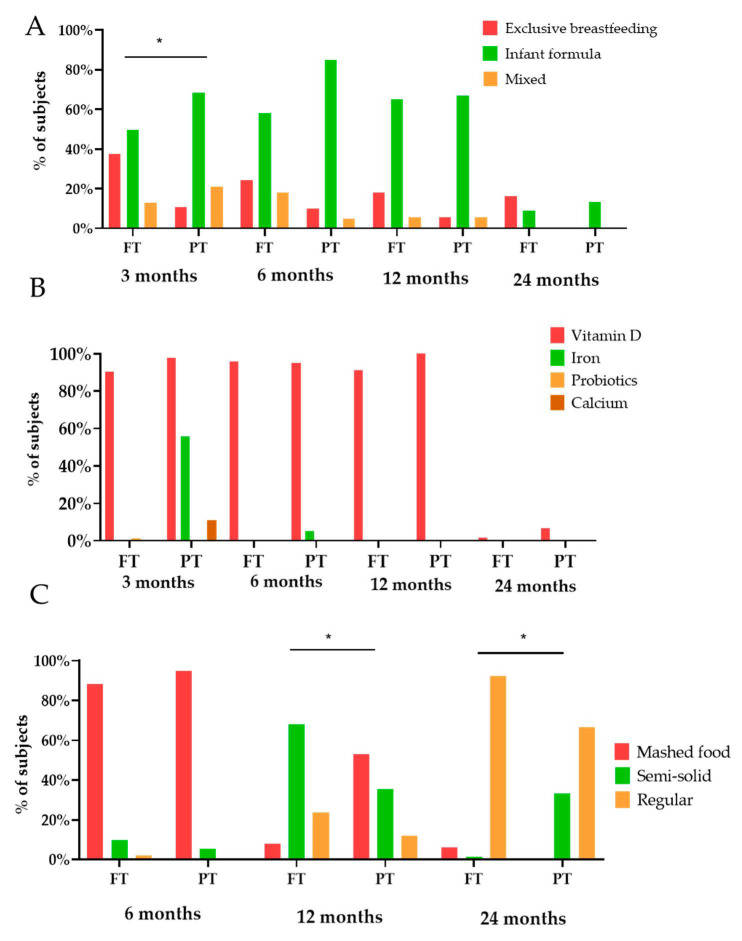
Differences between full-term (FT) and preterm (PT) infants regarding dietary characteristics ((**A**) type of lactation, (**B**) type of suplementatition and (**C**) food texture), according to age. Fisher’s test was used for intra-group comparisons. * *p* < 0.05.

The general characteristics of the feeding method related to lactation, the use of supplements or the texture of the food are presented in Figure 3. The percentage of breastfed infants was lower in PTs at 3 months of age.

Concerning the evolution in food texture (Figure 3C), at 6 months almost the totality of the sample consumed a pureed diet, incorporating solid food at 12 months and achieving a diet similar to the family diet at 2 years of age.

The differences in the intake of the major food groups between PT and FT at 6, 12 and 24 months of age are analyzed in Table 2. Concerning the timing of food introduction, at 6 months of age, the percentage of breastfed PT children is lower than that of FT (42.1% vs. 15%, respectively), contrary to what occurs with the consumption of processed infant cereals (56.8% in FT and 90% in PT). These differences between the two age groups studied disappear at 12 months of age. Interestingly, at 24 months, there is a lower percentage of preterm infants consuming fruit but a higher percentage of infant puree and infant processed foods. Regarding food intake, no variation was shown in any of the food groups analyzed at 6 months of age, except for potatoes and tubers, which had lower intake in the PT. While a higher intake of fruit, dairy and dairy products and meat and meat products was observed in the PT at 12 months, a lower intake of eggs and a higher intake of infant cereals was noted at 24 months.

### 3.3. Nutritional Targets in Children

With the aim to explore the existence of differences in the intake of energy, macro- and micronutrient between PT and FT from 6 to 24 months of age the analysis presented in Table 3 was conducted. It has been revealed that at 6 months, the intake of total fat and vitamin B_12_ was higher in PT, whereas at 12 months, animal protein intake was higher in PT than FT.

**Figure 4:**
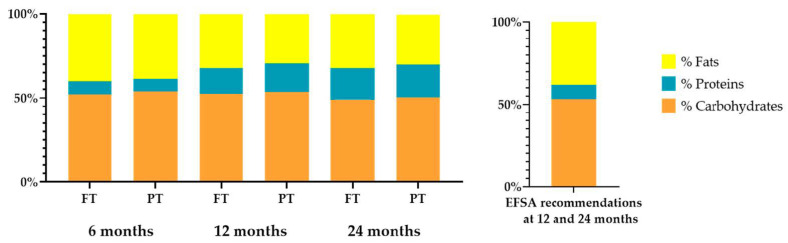
Percentage of the total energy intake provided by each macronutrient between full-term (FT) and preterm (PT) toddlers.

A moderate excess in protein intake was observed in both groups at 12 and 24 months, both in absolute amounts and relative to total energy intake (Figure 4). No significant differences in energy, macro- and micronutrients were observed in the analysis at 24 months between PT and FT.

Dietary vitamin D intake was highly compromised in both groups at all study times (at 6 months 10% and 17.2%, 12 months 0 and 0% and 24 months 0 and 1.5% of preterm and term infant met the EFSA AI recommendations). At 6 months, calcium, iron and zinc did not meet EFSA recommendations in any of the studied groups (25% and 28.4%, 80% and 74.7% and 100% and 93.4%, respectively). While recommendations for copper and selenium were only met by 13.1% and 11.5% in the FT group, they were met by 0% in both cases in the case of PT (data not shown).

**Figure 5:**
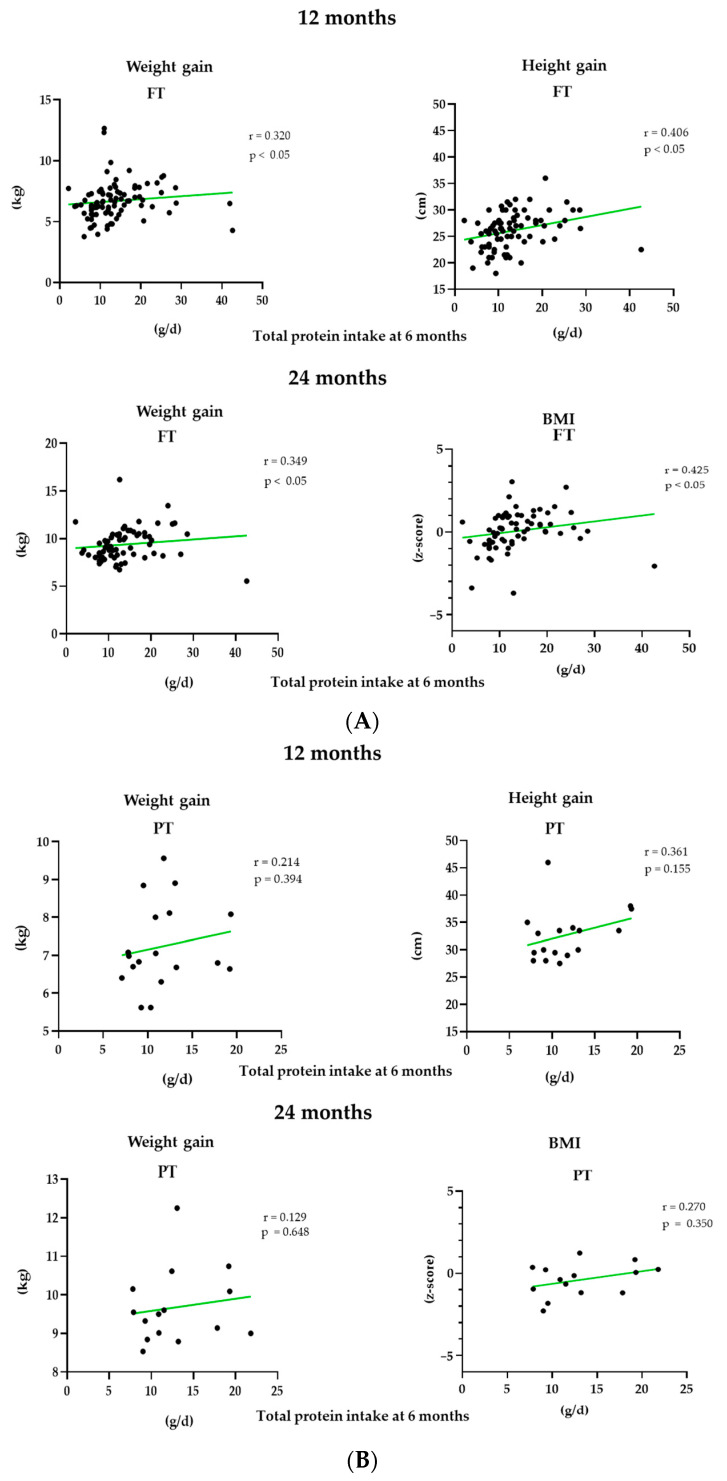
Spearman correlation in FT infants (**A**) and PT (**B**) between total protein intake at 6 months (g/d) and the different anthropometric parameters weight and height gain and BMI z-score at 12 and 24 months.

The association of protein intake at 6 months with anthropometric parameters at 12 and 24 months of age was further explored using Spearman correlation models in Figure 5. Positive associations were found between protein intake and weight and height gain at 12 and weight gain and BMI z-score at 24 months in the FT group. No significant differences were found in the PT group.

### 3.4. Bioactive Compounds

The average intake of the major classes and subclasses of (poly)phenols and fiber is presented in Table 4. Total phenols and total fiber intake in the sample were not different between groups at any studied time, with the exception of a higher intake of dihydrochalcones and soluble fiber in PT at 12 months.

## 4. Discussion

Information regarding complementary feeding patterns is of particular interest to better understand the impact of diet on health at early stages of life. In this context, these data provide an insight into the progressive food incorporation in a Spanish infant cohort from 6 to 24 months of age, allowing comparison between PT and FT infants and identifying the nutrients of particular interest in this population group.

According to previous studies, a higher percentage of first-time mothers, with some declared pathology during pregnancy and delivered by cesarean section, were found among PT groups [35]. In addition, as was expected due to the limitations widely described in initiation and maintenance of breastfeeding in PT [36], higher differences were detected in the prevalence of breastfeeding between the groups analysed. In contrast to the 42% of the FT newborns in the sample continuing to be breastfed up to 6 months of age, in compliance with WHO recommendations, less than 15% of PT maintained it [37]. However, since these recommendations are intended for healthy full-term infants, it is debatable whether they can be directly extrapolated to preterm infants since non-fortified exclusive breast milk may not cover the needs of these babies [38]. The nutritional depletion that some of them may have together with increased organ immaturity may increase the need for some key nutrients and energy, making supplementation or fortification strategies necessary [39,40]. Although one of the concerns of the scientific community is to identify the optimal time for the initiation of complementary feeding in PT babies, the observational nature of this work does not allow us to answer to this question. Some authors have proposed the age range of 5–8 months, of uncorrected age, as the recommended time for weaning in PT infants to allow adequate psychomotor and sensory development associated with solid food intake [41]. Like some other Mediterranean populations and developed countries in our cohort, we found 6 months of uncorrected age as the initial stage of weaning diet since almost 50% of PT children have begun to consume fruits, vegetables, tubers and meats by that time [13,42,43]. Nevertheless, in contrast to studies showing an early onset of food consumption of high dense energy foods in PT [44], we did not observe notable differences in comparison to FT infants. It is possible that some of these discrepancies may be explained by the use of corrected vs. chronological age, by the exclusion of extremely preterm infants in the analyses or by the reduced sample size in PT. Since in this study, only healthy infants born after 32 gestational weeks were enrolled, it is possible that the weaning recommendations for term infants could be used [41]. However, it should be noted that these data may not be extrapolable to extremely preterm infants. Over time, the amount of milk and formula consumed was reduced in favor of the introduction of new foods in the diet. Subsequently, from 6 to 12 months, legumes, eggs and dairy products were introduced in PT and FT babies, getting closer to the composition and texture of the family diet [45]. From a quantitative point of view, the consumption of potatoes and tubers at 6 months was significantly higher in FT infants. Results also suggest a higher consumption of cereals, infant cereals, vegetables and legumes in FT, while not reaching statistical significance, possibly due to the limited sample size. These differences disappear over time, except for higher intakes of fruit and infant cereals in PT infants at 12 and 24 months, respectively. At a nutritional level, energy intake was similar to that of term infants, increased directly with age and was adequate according to recommendations in all periods of time [46]. As far as the quantification of energy from breast milk is concerned, it is necessary to mention a limitation of the study. Since it was not possible to record the exact volume of milk produced by the mother, an indirect estimation was made using the average quantities established in the literature for each age range [24,25]. In addition, the intake of some essential micronutrients such as iron, zinc, vitamin D, vitamin B_12_ and folate are of particular interest in preterm babies in order to guarantee growth and development [7,8,9,10,11,12,13,14,15,16,17,18,19,20,21,22,23,24,25,26,27,28,29,30,31,32,33,34,35,36,37,38,39,40,41,42,43,44,45,46,47,48,49]. Based on the premise that the recommended daily intakes have been designed to reduce the risk of nutritional deficiency diseases in healthy babies, it can be inferred that the sample analyzed has a diet with an adequate nutritional intake for most nutrients. In this regard, vitamin D is a notable exception since it falls below the recommended values in almost the entire sample. Since most children (91.1 and 100% FT and PT respectively) receive supplementation of this vitamin up to 12 months of age and have adequate sun exposure, residing in a country with sufficient daylight hours, it is likely that the deficit of this vitamin from the diet is compensated [50].

From a qualitative perspective, the contribution of protein to energy intake at 12 and 24 months exceeded the recommendations in both groups of children analyzed, as is common in westernized countries, reaching intakes of 20% of total energy intake [51,52]. There is some discrepancy in the available literature regarding the impact of high protein intake during complementary feeding on weight gain, growth [53] and obesity risk [52,54,55]. In agreement with some authors who have hypothesized that the administration of a high protein diet between 6 and 12 months of age is related to greater weight gain during infancy, a direct correlation with weight gain at 12 and 24 months and height at 12 months has been found in FT infants. Thus, this paper adds to the existing information the finding of a non-association between high protein intake and growth or weight gain in preterm infants.

Finally, the information derived from the intake of bioactive compounds in the sample revealed some interesting results. Higher intakes of flavanols, flavones or soluble fiber in PT have been associated in adults with an increase in the gut microbiota of some microbial genera such as *Bifidobacterium* and *Lactobacillus*, which could have a beneficial impact on the maintenance of gut homeostasis in these children [56,57,58,59]. However, no data are available on the interaction between these compounds and the microbiota in early life. In addition to this prebiotic effect, birth involves a change in the oxidative balance, since there is a transition from a low-oxygen atmosphere to an oxygen-rich environment. Some authors have demonstrated that the intake of antioxidants from breast milk or from some foods, such as fruits and vegetables, in the perinatal stage can have a beneficial impact by counteracting the excess formation of oxygen-free radicals in children born prematurely. According to them, the higher intake of fruits in PT at 12 months of age, and therefore the higher levels of dihydrochalcones in this group, could be of great interest for their antioxidant properties in the prevention of some pathologies related to oxidative stress [60,61,62]. Future work studying the impact of the intake of these compounds during complementary feeding on oxidative stress biomarkers in PT infants could be useful to test this hypothesis.

## 5. Conclusions

This work is the first approach to examine complementary feeding practices in Spanish preterm infants. Our results allow us to suggest that complementary feeding is not different between preterm and full-term infants. Due to the different nutritional requirements between the groups, it seems necessary to design specific recommendations to cover and not exceed these requirements in preterm. Finally, studies specifically designed to analyze bioactive compounds in preterm children and their impact on gut microbiota and oxidative balance are needed.

## Figures and Tables

**Table 1:** General characteristics of the study sample.

Infant Factors		FT	PT
Sex (N 95) (N 20)	Male	54 (56.8)	8 (40.0)
	Female	41 (43.2)	12 (60.0)
Weight for age z-score at birth (N 93) (N 19)		0.00 ± 0.99	−4.66 ± 0.74 *
Height for age z-score (N 86) (N 16)		0.13 ± 0.91	−4.26 ± 1.40 *
BMI z-score at birth (N 86) (N 16)	Severe underweight (<−3)	2 (2.3)	15 (93.8) *
	Underweight (−3 to >−1)	16 (18.6)	0
	Normal weight (−1 to 1)	59 (68.6)	1 (6.3)
	Overweight (>1 to 2)	7 (8.1)	0
	Obese >2	2 (2.3)	0
Maternal factors			
Age (year) (N 87) (N 19)		34.45 ± 5.02	34.58 ± 3.75
Education level (N 94) (N 20)	Primary	4 (4.3)	2 (7.1)
	Secondary	13 (13.8)	1 (5.0)
	High school	26 (27.7)	7 (35.0)
	University	51 (54.3)	12 (60.0)
Weight pre-pregnancy (kg) (N 94) (N 20)		65.06 ± 12.29	64.64 ± 12.42
Weight at delivery (kg) (N 93) (N 18)		76.57 ± 11.06	68.98 ± 18.07
Height (cm) (N 94) (N 19)		1.64 ± 0.06	1.62 ± 0.05
Pre-pregnancy BMI (kg/m^2^) (N 94) (N 19)	Underweight (<18.5)	2 (2.1)	0
	Normal weight (18.5–25)	64 (68.1)	15 (78.9)
	Pre-obese (>25)	20 (21.3)	2 (10.5)
	Obese (>30)	8 (8.5)	2 (10.5)
Chronic diseases (N 13) (N 20)	Hypothyroidism	4 (30.8)	0
	Asthma	3 (23.1)	0
Pregnancy diseases (N 93) (N 20)	Diabetes	15 (16.1)	0 *
	Preeclampsia	4 (4.3)	3 (15.0)
	HT	3 (3.2)	5 (25.0) *
	Urinary infection	10 (10.8)	6 (30.0) *
	Gestational hypothyroidism	11 (11.82)	0
Pregnancy smoking status (N 94) (N 20)	Non-smokers	79 (84.0)	16 (80.0)
	Not during pregnancy	7 (7.4)	3 (15.0)
	Yes	8 (8.5)	1 (5.0)
Drugs intrapartum (N 94) (N 20)	Antibiotic	23 (24.5)	4 (20.0)
	Corticosteroids	0	4 (20.0) *
Delivery			
Type (N 94) (N 20)	Vaginal	73 (77.7)	12 (60.0)
	Cesarean section	21 (22.3)	8 (40)
Parity, n (N 94) (N 20)	0	48 (51.1)	17 (85.0) *
	1	38 (40.4)	2 (10.0)
	2	8 (8.5)	1 (5.0)

Data expressed as N (%) or mean ± standard deviation. FT, full-term. HT, hypertension. PT, preterm. Chi-square, Fisher and U-Mann–Whitney tests were used for intra-group comparisons. * *p* < 0.05.

**Table 2:** Differences in food consumption between Full-term (FT) and Preterm (PT) toddlers from 6 to 24 months.

	6 Months				12 Months				24 Months			
	FT (N 95)		PT (N 20)		FT (N 89)		PT (N 18)		FT (N 68)		PT (N 15)	
	%	Mean ± SD	%	Mean ± SD	%	Mean ± SD	%	Mean ± SD	%	Mean ± SD	%	Mean ± SD
Oils (g/d)	36.8	9.75 ± 1.45	25.0	10.00	97.8	10.49 ± 3.49	94.4	10.59 ± 2.43	98.5	12.45 ± 5.76	100	11.33 ± 3.52
Fruits (g/d)	56.8	261.18 ± 140.18	65.0	263.25 ± 92.97	94.4	270.93 ± 160.23	83.3	352.86 ± 110.47 *	97.1	222.37 ± 145.70	80 ^†^	257.51 ± 162.60
Vegetables (g/d)	44.2	159.90 ± 183.48	40.0	69.05 ± 33.24	98.9	145.51 ± 100.00	100	143.29 ± 83.28	94.0	91.53 ± 92.67	86.7	111.78 ± 114.32
Potatoes and tubers (g/d)	42.1	75.74 ± 78.64	35.0	31.59 ± 26.93 *	95.5	43.32 ± 31.03	88.9	48.23 ± 20.16	100	20.54 ± 16.43	100	26.83 ± 23.31
Cereals and cereal products (g/d)	17.9	17.12 ± 38.63	5.0	1.42	95.5	44.16 ± 36.40	83.3	37.42 ± 23.23	100	70.52 ± 42.49	93.3	75.99 ± 32.83
Legumes (g/d)	4.2	20.36 ± 15.84	5.0	1.42	75.3	15.33 ± 13.86	66.7	16.74 ± 14.72	93.8	18.14 ± 18.60	100	27.71 ± 40.10
Milk and dairy products ^1^	1.1	20.00	0.0	-	83.0	174.35 ± 187.24	83.3	209.40 ± 132.95 *	97.1	386.69 ± 228.50	100	410.15 ± 164.45
Meat and meat products (g/d)	23.2	22.15 ± 21.45	40.0	15.08 ± 6.37	96.6	33.83 ± 26.76	100	49.03 ± 33.73 *	100	35.58 ± 19.88	100	43.45 ± 23.58
Fish (g/d)	7.4	15.10 ± 17.76	0.0	-	93.3	33.53 ± 29.30	88.9	42.16 ± 37.33	92.6	39.19 ± 30.95	100	47.54 ± 44.18
Egg (g/d)	1.0	18.29	0.0	-	84.3	16.93 ± 10.79	83.3	13.41 ± 4.72	82.4	21.69 ± 8.44	93.3	16.96 ± 6.06 *
Breast milk (ml/d)	42.1	635.62 ± 217.47	15.0 ^†^	740.00 ± 69.28	21.3	545.26 ± 113.84	11.1	450.00 ± 212.13		-		-
Infant products	81.1	615.25 ± 402.13	95.0	600.35 ± 267.13	87.6	425.49 ± 236.12	94.4	366.91 ± 215.32	57.6	108.62 ± 190.10	86.7 ^†^	94.11 ± 95.87
Infant formula (mL)	74.7	628.95 ± 390.08	90.0	613.06 ± 241.79	70.8	469.52 ± 139.47	72.2	419.19 ± 139.38	8.8	491.67 ± 180.05	13.3	225.00 ± 21.21
Infant cereal (g/d)	56.8	16.76 ± 13.44	90.0 ^†^	13.21 ± 10.65	71.9	22.34 ± 11.04	88.9	20.25 ± 7.53	43.1	16.28 ± 10.68	66.7	24.35 ± 9.91 *
Infant puree (g/d)	17.9	105.91 ± 87.11	15.0	50.83 ± 30.21	21.3	114.02 ± 110.39	27.8	85.14 ± 59.37	2.9	98.22 ± 37.88	20 ^†^	45.24 ± 22.96
Sweets and desserts	8.4	3.75 ± 2.42	5.0	0.85	67.4	18.37 ± 19.98	55.6	15.41 ± 21.24	82.4	25.12 ± 23.88	60	27.25 ± 20.67

Data expressed as mean ± standard deviation—not available. ^1^ Milk and dairy products are represented as follows: milk (mL/day), yogurt and cheeses (g/day). U-Mann–Whitney test and Chi-square were used for intra-group comparisons. ^†^ Statistically significant differences in percentage of consumption. * Statistically significant differences in mean intake *p* < 0.05.

**Table 3:** Differences in the nutritional intake between Full-term (FT) and Preterm (PT) toddlers from 6 to 24 months.

	6 Months				12 Months				24 Months			
	FT (N 95)		PT (N 20)		FT (N 89)		PT (N 18)		FT (N 68)		PT (N 15)	
	%	Mean ± SD	%	Mean ± SD	%	Mean ± SD	%	Mean ± SD	%	Mean ± SD	%	Mean ± SD
Energy (kcal/d)	100	711.59 ± 261.47	100	639.97 ± 195.20	100	1067.68 ± 213.93	100	1082.43 ± 257.70	100	1123.22 ± 280.28	100	1189.88 ± 351.51
Macronutrients												
Fat (g/d)	100	31.31 ± 11.35	100	27.14 ± 9.54 *	100	37.74 ± 7.79	100	35.03 ± 5.41	100	39.91 ± 11.66	100	38.93 ± 5.74
SFA (g/d)	86.3	8.19 ± 6.21	95	7.86 ± 4.62	100	10.28 ± 5.05	100	10.08 ± 3.56	100	14.35 ± 5.65	100	14.23 ± 3.44
MUFA (g/d)	64.2	6.34 ± 6.04	60	5.38 ± 7.40	100	10.70 ± 3.47	100	11.36 ± 3.61	100	16.67 ± 5.36	100	15.80 ± 2.68
PUFA (g/d)	70.5	1.80 ± 2.80	75	1.37 ± 2.24	100	3.17 ± 1.82	100	3.27 ± 0.97	100	4.34 ± 1.32	100	4.61 ± 1.22
Carbohydrate (g/d)	100	92.63 ± 38.36	100	86.05 ± 29.91	100	139.92 ± 31.37	100	144.88 ± 52.07	100	137.67 ± 42.48	100	150.17 ± 65.40
Dietary fiber (g/d)	78.9	7.40 ± 5.74	95	5.27 ± 4.00	100	14.81 ± 5.14	100	17.58 ± 10.08	100	15.26 ± 5.68	100	19.55 ± 12.85
Protein (g/d)	100	13.62 ± 7.16	100	12.34 ± 4.29	100	41.05 ± 14.87	100	45.64 ± 16.18	100	52.76 ± 15.33	100	58.30 ± 18.23
Animal protein (g/d)	31.6	4.23 ± 4.94	40	3.16 ± 1.29	100	19.99 ± 12.34	100	26.09 ± 13.36 *	100	32.88 ± 12.40	100	35.40 ± 10.41
Vegetal protein (g/d)	65.3	4.00 ± 3.41	75	2.25 ± 1.30	100	12.86 ± 5.23	100	12.72 ± 7.02	100	17.42 ± 6.79	100	19.55 ± 13.78
Micronutrients												
Vitamin D (μg/d)	100	6.89 ± 3.64	100	6.99 ± 2.89	100	6.24 ± 2.78	100	6.64 ± 2.56	100	3.13 ± 3.36	100	3.56 ± 2.08
Vitamin E (mg/d)	100	7.73 ± 4.71	100	8.09 ± 3.32	100	9.92 ± 5.19	100	9.79 ± 3.40	100	5.65 ± 2.64	100	6.48 ± 2.87
Folate (μg/d)	64.2	94.85 ± 96.68	75	68.07 ± 41.01	100	427.57 ± 209.95	100	546.95 ± 409.12	100	497.23 ± 208.35	100	768.83 ± 184.91
Vitamin B_12_ (μg/d)	100	2.33 ± 1.29	100	1.63 ± 1.11 *	100	2.74 ± 1.16	100	2.68 ± 1.41	100	3.24 ± 1.67	100	3.16 ± 1.40
Calcium (mg/d)	100	421.37 ± 194.41	100	397.29 ± 189.51	100	678.72 ± 248.89	100	698.48 ± 172.74	100	726.71 ± 293.68	100	766.56 ± 184.91
Selenium (μg/d)	64.2	5.19 ± 9.40	75	3.10 ± 2.64	100	39.60 ± 20.12	100	42.82 ± 26.14	100	65.98 ± 22.60	100	73.07 ± 22.74
Iron (mg/d)	100	6.25 ± 3.26	100	5.70 ± 2.74	100	10.19 ± 2.90	100	10.97 ± 4.09	100	7.80 ± 3.12	100	10.47 ± 6.02
Copper (mg/d)	64.2	0.21 ± 0.16	75	0.16 ± 0.09	100	0.69 ± 0.30	100	0.82 ± 0.54	100	0.91 ± 0.32	100	1.20 ± 0.72
Zinc (mg/d)	100	3.89 ± 2.35	100	3.83 ± 2.00	100	6.32 ± 1.97	100	6.90 ± 1.68	100	6.63 ± 2.20	100	7.40 ± 2.92

ALA, alpha-linolenic acid. DHA, docosahexaenoic acid. FT, full-term. PT, preterm. LA, linoleic acid. MUFA, monounsaturated fatty acids. PUFA, polyunsaturated fatty acids. SFA, saturated fatty acids U-Mann–Whitney test was used for intra-group comparisons. * *p* < 0.05.

**Table 4:** Differences in the intake of bioactive compounds (phenols and fibers) between Full-term (FT) and Preterm (PT) from 6 to 24 months.

	6 Months				12 Months				24 Months			
	FT (N 95)		PT (N 20)		FT (N 89)		PT (N 18)		FT (N 68)		PT (N 15)	
Bioactive Compounds	%	Mean ± SD	%	Mean ± SD	%	Mean ± SD	%	Mean ± SD	%	Mean ± SD	%	Mean ± SD
Total phenols (mg/d)	64.2	373.55 ± 254.14	75.0	369.46 ± 216.83	100	702.77 ± 347.54	100	896.36 ± 703.85	100	764.05 ± 361.12	100	834.57 ± 628.02
Total (poly)phenols (mg/d) ^1^	65.3	142.50 ± 99.97	75.0	126.35 ± 72.60	100	134.16 ± 73.44	100	191.02 ± 161.90	100	127.33 ± 84.92	100	100.47 ± 78.43
Flavonoids (mg/d)	65.3	104.48 ± 78.26	75.0	102.54 ± 57.54	100	89.48 ± 55.96	100	131.00 ± 120.10	100	85.23 ± 68.84	100	66.07 ± 58.23
Anthocyanins	56.8	12.90 ± 8.18	70.0	11.61 ± 7.91	95.6	24.94 ± 27.58	88.8	44.20 ± 47.34	100	28.28 ± 32.78	86.7	23.74 ± 26.25
Dihydrochalcones	53.7	1.56 ± 0.97	60.0	1.53 ± 0.42	88.8	1.48 ± 1.22	77.8	2.25 ± 1.91 *	82.4	1.29 ± 1.25	60	0.93 ± 1.02
Flavanols	64.2	74.07 ± 57.84	75.0	67.95 ± 40.11	100	44.32 ± 31.81	100	62.19 ± 57.00	100	38.93 ± 32.57	100	27.90 ± 27.77
Flavanones	58.9	20.98 ± 22.77	75.0	22.47 ± 18.39	96.6	19.78 ± 20.98	100	27.14 ± 42.38	100	15.78 ± 22.80	86.7	18.03 ± 21.15
Flavones	64.2	0.14 ± 0.24	75.0	0.05 ± 0.14 *	100	0.56 ± 0.56	100	0.50 ± 0.40	100	0.53 ± 0.57	100	0.77 ± 0.98
Flavonols	64.2	6.86 ± 5.83	75.0	5.84 ± 4.36	100	19.96 ± 23.91	100	25.54 ± 32.79	100	18.13 ± 20.07	100	18.30 ± 21.68
Isoflavanoids	65.3	0.01 ± 0.01	75.0	0.00 ± 0.00 *	100	0.34 ± 1.06	100	0.13 ± 0.18	100	0.65 ± 1.38	100	0.64 ± 0.63
Phenolic acids (mg/d)	63.2	35.93 ± 28.73	70.0	25.15 ± 15.71	100	43.38 ± 21.77	100	58.88 ± 42.11	100	40.08 ± 20.27	100	32.30 ± 21.66
Lignans (mg/d)	29.5	5.49 ± 11.30	10.0	1.13 ± 1.14	92.1	0.11 ± 0.15	100	0.13 ± 0.15	94.1	0.16 ± 0.20	100	0.15 ± 0.12
Non-phenolic metabolites (mg/d)	64.2	0.25 ± 0.31	75.0	0.20 ± 0.14	100	1.18 ± 0.60	100	1.52 ± 0.84	100	1.77 ± 0.56	100	2.07 ± 0.70
Other polyphenols (mg/d)	23.2	2.15 ± 1.15	5.0	2.75	84.3	1.43 ± 2.17	88.8	1.12 ± 1.36	91.1	2.06 ± 3.29	93.3	2.09 ± 2.63
Dietary fiber (g/d)	78.9	7.40 ± 5.74	95.0	5.25 ± 4.00	100	14.81 ± 5.12	100	17.58 ± 10.08	100	15.26 ± 5.68	100	19.55 ± 12.85
Insoluble	65.3	4.94 ± 3.34	75.0	4.10 ± 2.17	100	9.68 ± 4.22	100	11.74 ± 8.17	100	9.73 ± 3.93	100	11.59 ± 8.32
Soluble	66.3	1.47 ± 1.11	80.0	1.26 ± 1.23	100	2.15 ± 0.91	100	2.66 ± 1.26 *	100	1.70 ± 0.67	100	2.82 ± 1.40 *

Values are presented as mean ± standard deviation. ^1^ Total polyphenols were calculated as the sum of total flavonoids, phenolic acid, lignans, non-phenolic metabolites and other polyphenols. U-Mann–Whitney test and Chi-square were used for intra-group comparisons. * *p* < 0.05.
